# Emergence of Catalytic Activity in VRK3: Phosphoproteomic Insights into the Regulatory Network of a Former Pseudokinase

**DOI:** 10.3390/proteomes14010014

**Published:** 2026-03-18

**Authors:** Ayadathil Sujina, Amal Fahma, Suhail Subair, Rajesh Raju, Poornima Ramesh

**Affiliations:** Centre for Integrative Omics Data Science (CIODS), Yenepoya (Deemed to be University), Mangalore 575018, Karnataka, India; sujinaa.ciods@yenepoya.edu.in (A.S.); amalfahmaak.ciods@yenepoya.edu.in (A.F.); suhailsubair.ciods@yenepoya.edu.in (S.S.)

**Keywords:** phosphoproteomics, VRK3, kinase, substrate, protein–protein interaction, signaling pathway

## Abstract

Vaccinia-Related Kinase 3 (VRK3) is increasingly recognized as a crucial signaling modulator in both normal and pathological processes. This kinase was long thought of as a catalytically inactive pseudokinase, until recently it was established to phosphorylate Barrier to Autointegration Factor (BAF) proteins through its extracatalytic domain. VRK3 regulates diverse cellular pathways through scaffold interactions and context-dependent phosphorylation. This review is centered around the phosphoregulatory network that modulates VRK3 phosphorylation with implications in its abundance and function. A large-scale phosphoproteomic data integration was performed by combining phosphoproteomics profiling and differential phosphorylation from 115 mass spectrometry studies, identifying 32 high-confidence phosphorylation sites on VRK3. Notably, VRK3 (S59), (S82), and (S83) were predominantly observed highlighting plausible functional significance. These phosphorylation sites share 33 potential upstream kinases, and multiple interactor proteins, which in combination are known to regulate ERK, Hippo, and GPCR pathways. These insights advance the understanding of phosphorylation control by kinases and highlight opportunities to target VRK3-associated networks for therapeutic intervention in diseases such as glioma and liver cancer.

## 1. Introduction

Vaccinia-Related Kinase 3 (VRK3) is a member of the VRK family of serine/threonine protein kinases, including VRK1, VRK2, and VRK3. Through initial structural elucidations, VRK3 contained critical amino acid substitutions in its kinase domain, was found to lack ATP binding sites, and significant changes in active site conformation [1,2]. Therefore, it was categorized as a true pseudokinase with evolutionary significance [3]. Later studies showed that the N-terminal extracatalytic domain (amino acid 1-147), along with the kinase domain (showing high sequence similarity to VRK1 and VRK2) mediated the phosphorylation of Barrier to Autointegration Factor (BAF) [4]. Additionally, VRK3 plays important roles as a regulatory scaffold protein and signaling mediator in diverse cellular processes [5].

Structurally, the canonical isoform of human VRK3 is 474 amino acids in length with the kinase domain spanning the C-terminal. The N-terminal region contains extracatalytic domain with a bipartite nuclear localization signal that ensures its nuclear distribution [4,5]. Functionally, VRK3 has been implicated in cell cycle regulation and chromatin remodeling through interactions with other nuclear proteins. Notably, it also associates with proteins essential for chromatin assembly, cell-cycle progression, and nuclear envelope dynamics, including the chromatin remodeler BAF [4,6,7]. Acting primarily as a scaffold, VRK3 binds partners in the Extracellular signal-Regulated Kinase (ERK) signaling pathway and stabilizes the vaccinia H1-related (VHR) or DUSP3 phosphatase, thereby modulating MAPK/ERK activity independently of kinase function [1,8,9]. Emerging evidence also links VRK3 to neurodevelopmental disorders. In animal models, VRK3 deletion leads to autism-like behaviors and structural abnormalities in the hippocampus, suggesting a role in synaptic development and plasticity [10]. Additionally, elevated VRK3 abundance in cancerous tissues has been associated with altered cell cycle progression, particularly in liver cancer, highlighting potential relevance in oncogenesis [6].

Despite such established roles, VRK3 remains insufficiently characterized through a phosphoproteomics perspective, with scant information available concerning its co-regulating partners, upstream kinases, and interacting partners. In this review article, this substantial knowledge gap in the regulatory circuitry associated with canonical proteoform of VRK3 is systematically examined through extensive phosphoproteomics data integration and harmonization. The phosphorylation dynamics were explored through canonical proteoforms of respective upstream kinases, interacting proteins, and co-differentially abundant proteins that display conserved differential abundance patterns across multiple cellular phosphoproteomic datasets. By implementing reproducible and methodologically rigorous data integration and harmonization, the study delineates novel insights into phosphoproteomic landscape of VRK3 and highlights the growing importance of proteins as regulatory centers in health and illness.

## 2. VRK Family: Vaccinia-Related Kinase Family

The vaccinia-related kinases (VRKs; named after their homology to vaccinia virus B1 kinase) form a small family of mammalian Serine/Threonine protein kinases, including VRK1, VRK2, and VRK3. Despite structural similarities, they have distinct biochemical properties and biological roles. VRK1 is an active nuclear kinase that phosphorylates chromatin-associated substrates, including BAF/BANF1, regulating nuclear envelope dynamics, chromatin structure, and DNA damage responses [11,12]. It is essential for cell-cycle progression and proliferation, and its differential abundance is linked to therapy resistance in cancer [13,14]. Conversely, germline mutations are associated with motor-neuron and neurodevelopmental disorders, such as SMA-like disease and pontocerebellar hypoplasia type 1A (PCH 1A) [15,16]. VRK2, also an active kinase, exists in two isoforms: VRK2A, anchored to the endoplasmic reticulum and nuclear envelope via a transmembrane domain, and VRK2B, a soluble form present in the nucleoplasm and cytosol [17,18]. Functionally, VRK2 acts as a scaffold or modulator of MAPK signaling by interacting with JIP1 and KSR1-MEK1 complexes, thereby reducing JNK and ERK activity [19,20]. It can also phosphorylate BAF in certain contexts [12]. Genetic studies suggest the involvement of VRK2 in psychiatric disorders like schizophrenia and cancer [12]. Conversely, VRK3, long thought of as a pseudokinase, was recently established to regulate signaling via protein–protein interactions. Notably, it activates dual-specificity phosphatase VHR/DUSP3, which promotes ERK dephosphorylation and reduces ERK pathway activity [8,21]. Therefore, while VRK1 and VRK2 are catalytically active kinases involved in chromatin regulation, cell-cycle control, and signal transduction, VRK3 functions as a regulator that modulates ERK signaling, illustrating the functional diversity and clinical importance of this kinase family.

## 3. Phosphorylation Landscape of VRK3

**Figure 1:**
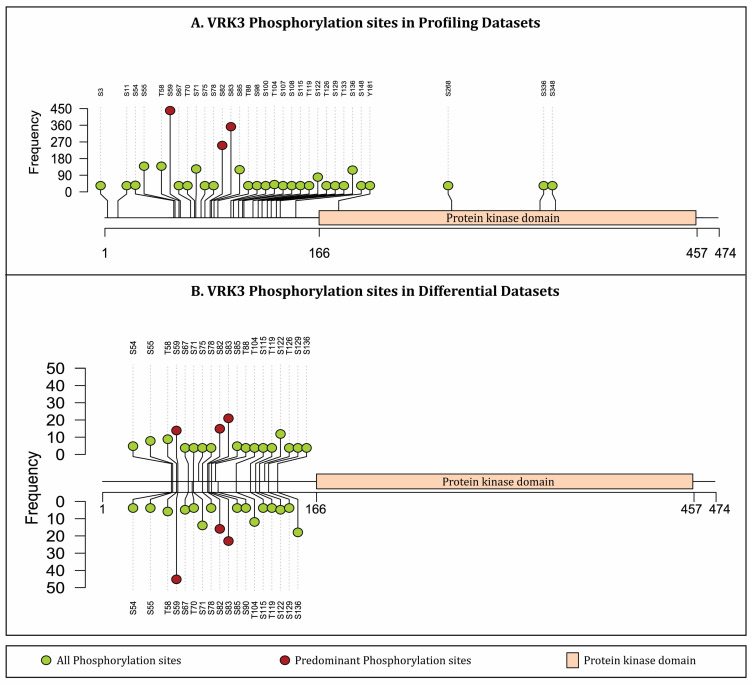
**Lollipop plot showing the frequency distribution of VRK3 phosphorylation sites obtained from profiling and differential datasets.** (**A**) VRK3 phosphorylation sites detected across 635 phosphoproteomics profiling datasets; (**B**) VRK3 phosphorylation sites detected across 142 datasets showing differential abundance of VRK3 phosphopeptides. Frequency indicates the number of datasets in which a particular phosphorylation site is identified/differentially abundant. R/Bioconductor package track Viewer was utilized for this representation.

Phosphoproteomics is a powerful platform utilized for large-scale profiling of phosphorylated proteins within a biological context and time point, instrumental in elucidating cellular signaling networks. Mass spectrometry-based techniques have facilitated the identification of multiple phosphorylation sites on VRK3 and its co-differentially abundant proteins, offering valuable insights into their functional roles. Several attempts have been previously made to integrate phosphoproteomics data that deduces VRK3 phosphorylation [22,23,24,25]. However, such attempts are prominently lacking in establishing a co-regulatory network of VRK3 with other proteins. Therefore, it is intriguing to integrate these datasets to decipher further functional analysis, protein interaction networks, and kinase-substrate prediction techniques. Interestingly, during the literature survey we found 3825 publicly available research articles describing mass spectrometry-based phosphoproteomics experiments (published between 2010 and 2025). The survey for appropriate datasets was conducted in June 2025 through PubMed database using the keywords “phosphoproteomics” OR “phosphoproteome” NOT “Plant” NOT “Review”. Post-survey, each publication was manually interrogated and evaluated for inclusion/exclusion as follows. Inclusion criteria: original research articles indexed in PubMed, studies reporting global, high-throughput phosphoproteomic analyses through mass spectrometry, human cell line-based experimental conditions, availability of phosphorylation site level data. The exclusion criteria were as follows: review articles, studies conducted exclusively in plants, mouse, rat, or other non-human model organism, studies without accessible or extractable phosphorylation site information. We systematically cataloged these studies to examine the phosphoproteomic landscape of VRK3. It is important to note that due to variability of analysis platforms, sample preparation protocols, and data analysis pipelines associated with mass spectrometry-based phosphoproteomics workflows, reanalysis of raw data from multiple studies is not feasible. Therefore, we cataloged the phosphorylation status of VRK3 and co-regulated phosphoproteins as reported in the original publications. As a result, 115 among 3825 studies (PMIDs/individual publications) specifically identified phosphorylation events of VRK3 through phosphopeptide identification and quantification. By considering protein phosphorylation at different time points under various biological and experimental conditions, data from115 studies was further categorized into 635 human cellular phosphoproteomics profiling datasets (Supplementary Table S1). In these datasets at least one phosphopeptide belonging to VRK3 was identified. Further, 142 datasets among the 635 showed differential phosphorylation of VRK3 through phosphopeptide quantification (Supplementary Table S2). A total of 32 high-confidence (≥75% or an A score ≥13) phosphorylation sites on VRK3 were identified from phosphoproteomics profiling datasets, among which, 21 phosphorylation sites of VRK3 were harbored on differentially abundant phosphopeptides (Figure 1). Notably, the phosphorylation sites S59, S82, and S83 were the most frequently cataloged sites in phosphoproteomic studies, showing consistent differential phosphorylation across diverse biological and experimental conditions. However, mere detection of predominant sites across studies does not imply phosphoregulation of VRK3, rather, it shows that in spite of drastic variability across biological conditions, experimental protocols, and data analysis pipelines, these sites were persistently detectable.

## 4. Functional Association of Phosphorylation Sites in Proteins Co-Differentially Abundant with Specific VRK3 Phosphorylation Sites

VRK3 phosphorylation predominantly at S59, S82, and S83, which are found outside the kinase domain [26] links VRK3 to an extensive network of proteins engaged in fundamental biological processes. Specifically, recent studies have utilized extensive bioinformatics to understand co-differential abundance of phosphorylation patterns among relevant groups of proteins, that are associated with specific functions [27,28,29]. Therefore, we listed proteins that showed conserved co-phosphorylation with VRK3 phosphorylation when compared across the datasets (Supplementary Table S3). Major co-abundant proteins include proteins such as PTPN2 (co-abundant in 16 studies), CDC20 (17), SRRM2 (181), SRSF2 (58), RIOK1 (47), FOXK2 (76), TAF4 (2), MACIR (33), ABI2 (47), and CKAP4 (15). Protein tyrosine phosphatase non-receptor type 2 (PTPN2) is a critical modulator of pro-inflammatory cytokine signaling, dephosphorylating and inhibiting JAKs and STATs while controlling TCR signaling through dephosphorylation of SFK activation motifs [30]. Cell division cycle protein 20 (CDC20) governs mitotic progression and ensures accurate chromosome segregation during late mitosis [31]. RIOK1 suppresses the p38 MAPK/PMK-1 pathway and is indispensable for the cytoplasmic maturation of the 40S ribosomal subunit during ribosome biogenesis [32]. SRRM2, a nuclear-speckle marker rich in intrinsically disordered domains, is implicated in a range of human diseases when dysfunctional [33]. Components of the TAF4 basal transcription factor complex are essential for initiating RNA polymerase II-dependent transcription [34].

Splicing regulators such as serine/arginine-rich splicing factor 2 (SRSF2) [35] and SF3B2 [36] play pivotal roles in mRNA processing and stability, while ABI2 participates in cytoskeletal remodeling, membrane ruffling, and cell migration [37]. FOXK2, a member of the forkhead box (FOX) transcription factor family, orchestrates diverse cellular activities such as cell-cycle control, apoptosis, differentiation, and metabolism [38,39]. Overall, these co-abundant proteins emphasize the diverse regulatory functions of VRK3 S59, S82, and S83 in signaling, transcription control, splicing, cytoskeletal dynamics, and cell cycle regulation.

## 5. Upstream Kinases with Potential to Modulate VRK3 Phosphorylation

**Figure 2:**
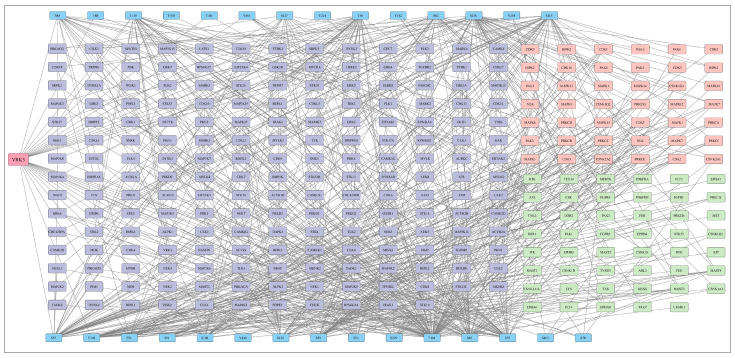
**Potential upstream kinase network of VRK3.** with blue nodes representing the phosphorylation sites of VRK3, violet nodes representing the upstream kinases analyzed through PSAP analysis, green nodes representing the predicted upstream kinase, and pink representing the common kinases from both predicted and PSAP analysis. The figure is generated using Cytoscape version 3.10.3.

As a prime scaffolding protein that phosphorylates BAF, VRK3 indirectly regulates ERK/MAPK pathway, also creating a negative feedback loop [4]. However, the phosphorylation of VRK3 itself remains to be completely elucidated. Till date, experimentally deciphered phosphorylation of VRK3 is known to be mediated only by Cyclin-Dependent Kinase 5 (CDK5). VRK3 and CDK5 work together to regulate neuroprotective mechanisms in response to glutamate-induced oxidative stress. This pathway limits neuronal cell death caused through apoptosis by reducing sustained activation of ERK1/2 [40]. Certain other potential upstream kinases of VRK3 are also predicted using tools including NetworKIN [41], AKID [42], and the in vitro Kinase to Phosphosite database (iKiP-DB) [43]. Furthermore, such kinases were also identified through synthetic peptide library screening (PSAP analysis) by Johnson et al. (2023), which provides semi-experimental leads towards VRK3 phosphorylation [44]. Such resources contain useful data which can be further substantiated with experimental results. The overall kinase regulatory network predicted by the above resources for all catalogued phosphorylation sites of VRK3 is represented in Figure 2; while specific upstream kinases of predominant VRK3 sites along with prediction score and algorithm are reported in Supplementary Table S4.

**Figure 3:**
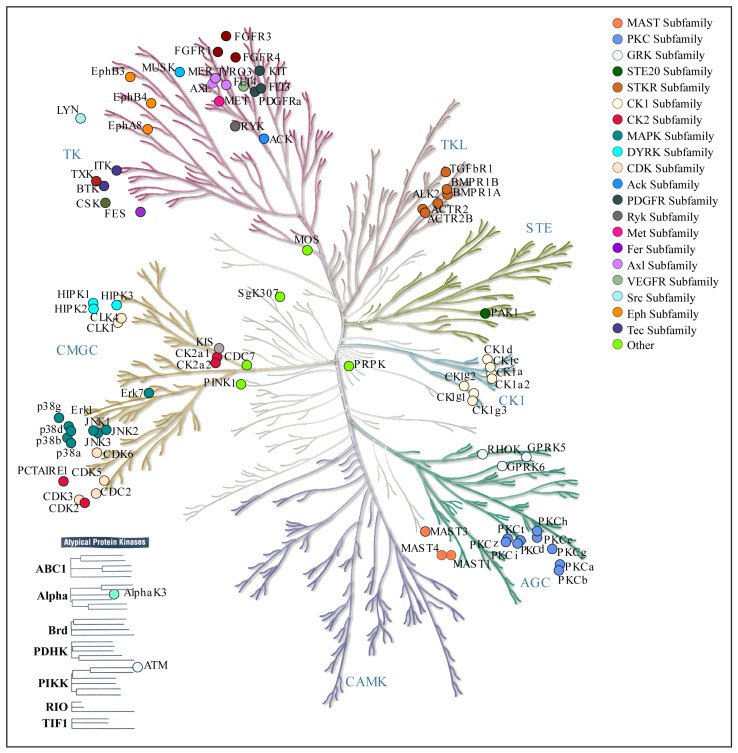
**Phylogenetic tree of potential upstream kinases of VRK3.** The map shows the classification of VRK3 kinases into major families based on sequence similarity. Distinct kinase groups such as AGC, CAMK, CMGC, TK, and TKL are color-coded. The map is generated through the KinMap online tool.

Among the potential upstream kinases of VRK3, 33 kinases were found to consistently co-differentially abundant with VRK3 phosphorylation. This overlap between computational predictions and experimental data strengthens the reliability of these kinases and highlights their likely biological importance in regulating VRK3-associated phosphorylation. The high agreement suggests that these kinases may form a core group of upstream regulators of VRK3. Primarily, kinases that modulate VRK3 include Tyrosine Kinases (TK), Tyrosine Kinase-Like (TKL), Sterile 20-like Kinases (STE), CMGC kinases (including CDK, MAPK, GSK, CLK families), Calcium/Calmodulin-Dependent Kinases (CAMK), Protein Kinase A, G, and C families, Casein Kinase 1, and Atypical Kinases (Figure 3).

Certain phosphorylation sites in these upstream kinases also showed co-differential abudance with VRK3 phosphorylation at predominant sites. For example, TGFBR2 (S548) and EEF2K (S445) showed co-differential abundance with VRK3 (S59); LATS1 (S464, S613) showed co-differential abundance with VRK3 (S82), and TTBK2 (S786) and GRK2 (S685) showed co-differential abundance with VRK3 (S83). The TGF-β type II receptor (TβR-II) is a transmembrane serine/threonine kinase that, upon ligand binding, recruits and phosphorylates another transmembrane kinase [45], while eukaryotic elongation factor 2 kinase (eEF2K) is an atypical protein kinase that inhibits the elongation phase of protein synthesis [46]. It also helps ribosomes move along mRNAs during translation [47,48]. TTBK2 is a serine/threonine kinase classified within the casein kinase 1 (CK1) family of eukaryotic protein kinases [49]. LATS1 functions as an inhibitor of YAP1 within the Hippo signaling pathway, which is crucial for regulating organ size and preventing tumor growth by limiting cell proliferation and promoting programmed cell death [50]. GRK2 influences heart function and development, including cardiac muscle contraction, regulation of smooth and striated muscles, and GPCR signaling pathways. It also plays roles in protein phosphorylation, receptor internalization, catecholamine secretion, and viral processes like host cell entry and genome replication [51]. Overall, phosphorylation of VRK3 by these kinases links signals from growth factor pathways (TGFBR2), translation and metabolic control (EEF2K), tumor suppression and organ size regulation (LATS1), cytoskeletal regulation (TTBK2), and GPCR-mediated signaling (GRK2). This points to VRK3 functioning as a multi-pathway integrator, operating at the intersection of various signaling networks to coordinate cellular responses to both environmental and internal stimuli.

## 6. Protein Interactors of VRK3 That Plausibly Modulate VRK3 Function

**Figure 4:**
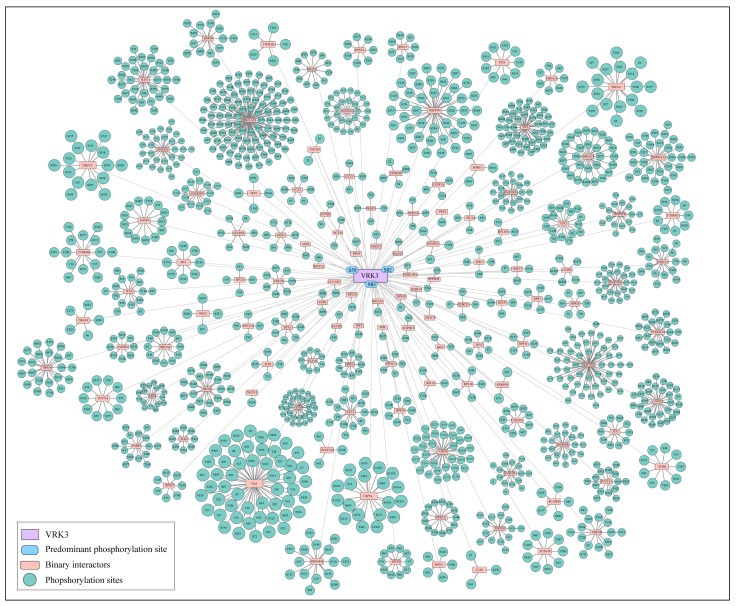
**Co-differential abundance of binary interactors of VRK3.** Certain proteins showed highly conserved co-phosphorylation patterns with VRK3 phosphorylation at S59, S82, and S83 under various physiological functions and experimental conditions. The figure is generated using Cytoscape version 3.10.3.

As VRK3 acts as a signaling scaffold in the cell, majority of its functions are modulated through protein–protein interactions. Important interactors including BAF and VHR/DUSP3, are also implicated in its kinase activity described in the previous sections. To further explore other putative interactors, the experimentally known protein–protein interactors of VRK3 were extracted from HPRD [52], BIND [53], BioGRID [54], ConsensusPathDb [55], CORUM [56], and RegPhos 2.0 [57]. Among the interactors, several phosphoproteins exhibited co-differential abundance with VRK3 phosphorylation; where PCYT1A, EMD, HSPA4, H1-4, RPLP2, VIM, NCL, FOXP1, and ATXN2 showed co-differential abundance with all three VRK3 predominant sites (Figure 4).

**Figure 5:**
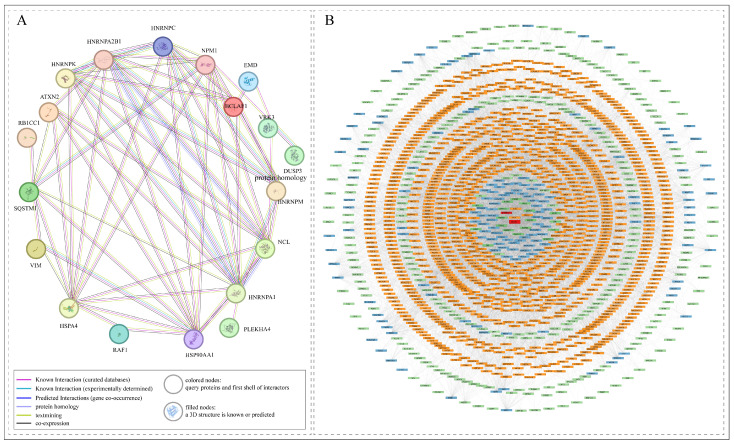
**Interaction networks of VRK3 and DUSP3.** (**A**) STRING-based interaction map depicting the primary interactors of VRK3 (interaction confidence threshold: 0.9). (**B**) Extended network of DUSP3 showing its direct and secondary interactors (interaction confidence threshold: 0.9, derived only from experimental and curated databases). The binary interactors of VRK3 (from panel **A**) are also highlighted within the DUSP3 network (red nodes), illustrating the overlapping interaction nodes (orange, blue and green nodes obtained by extending network through first, second and third shell of interactors) and potential shared signaling partners between VRK3 and DUSP3. Figure was generated using Cytoscape version 3.10.3.

Co-differential abundance analysis of the binary interactors of VRK3 revealed that several proteins exhibit highly conserved co-phosphorylation patterns with VRK3 at sites S59, S82, and S83 across diverse physiological and experimental conditions. These consistent phosphorylation trends suggest a coordinated regulatory mechanism, emphasizing the central role of VRK3 in modulating specific signaling events (Figure 4). STRING analysis (interaction confidence threshold: 0.9) identified a direct interaction between VRK3 and DUSP3, a dual-specificity phosphatase that regulates MAPK signaling, indicating a potential reciprocal regulatory relationship (Figure 5A) [58]. Although a direct interaction between VRK3 and DUSP3 was not detected in our dataset, several DUSP3-interacting proteins were found to be co-differentially abundant and interconnected with VRK3-associated proteins. These shared interactors collectively form an extended network linking VRK3 and DUSP3, suggesting an indirect regulatory relationship (interaction confidence threshold: 0.9, derived only from experimental and curated databases). Importantly, many of these interactors were also identified from our dataset, providing experimental support and reinforcing the reliability of the observed associations (Figure 5B). The integrated network derived from these findings highlights VRK3 as a phosphorylation-dependent signaling hub, potentially influencing DUSP3-mediated signaling through intermediary proteins and coordinating diverse cellular and stress-response pathways.

## 7. Functional Insights and Emerging Directions in VRK3-Linked Disorders

VRK3 is highly abundant during developmental stages and contributes to cell cycle progression by phosphorylating the nuclear envelope-associated protein BAF [10]. VRK3 also serves as a crucial regulator of ERK signaling by inhibiting ERK activity through direct interaction with the nuclear mitogen-activated protein kinase phosphatase, VHR/DUSP3, which deactivates ERK [8]. In the context of the nervous system, VRK3 is considered essential for neuronal function. The reported reduction in VRK3 in response to amyloid-β oligomers implicates it in Alzheimer’s disease, through synaptic dysfunction; however, these studies largely focuses on protein abundance than post translational modifications [59]. Additionally, VRK3 facilitates the nuclear translocation of glutamate-induced heat shock protein 70 (HSP70), implicating it in neuronal stress response pathways [9]. Under oxidative stress conditions, VRK3 is phosphorylated at S108 by CDK5, a modification that limits sustained ERK activation and prevents cell death, thereby underscoring its role in cellular protection and stress regulation [40]. Our phosphoproteomics data integration showed that S108 is infrequently phosphorylated in distinct experimental conditions compared to the predominant phosphosites, suggesting that CDK5 mediated phosphorylation of VRK3 may be involved in stress regulatory pathways.

VRK3, identified through a kinome-wide RNA interference screen, is essential for the survival of glioma cells harboring an H3K27M mutation. In addition to causing metabolic reprogramming, which shifts cells toward oxidative phosphorylation without increasing mitochondrial content, its silence causes G1-phase cell cycle arrest and disturbs chromatin condensation, as seen by decreased phosphorylation of histone H3 at S10 and S28. A poor prognosis in adult gliomas is clinically associated with high VRK3 abundance, and its selective lethality in cells with an H3K27M mutation indicates a tumor-specific treatment window that might protect healthy neural stem cells. According to these results, VRK3 is a potential and situation-specific target in the rapidly changing field of glioma treatments [7]. Importantly, existing studies have not examined whether distinct VRK3 phosphorylation states contribute to its tumor-specific functions, representing a critical gap that phosphoproteomic analyses could address, as evidenced by the dynamic upregulation of specific VRK3 phosphosites observed in tumor-specific datasets.

By controlling cell cycle phases, VRK3 contributes to the pathophysiology of liver cancer. Hepatocellular carcinoma tissues and cell lines showed elevated levels of VRK3, which, in contrast to VRK1, primarily controls the G1/S transition. Its knockdown caused cell cycle arrest at the S and G2/M phases. These effects seem to be mediated by interactions with important regulatory proteins, including centriolin (CNTRN) and SPIN1, which are involved in the regulation of mitotic spindle checkpoints and chromatin condensation, respectively. The results taken together suggest that VRK3 may be an oncogenic driver of liver cancer, possessing distinct cell cycle regulatory roles that could provide new therapeutic avenues [6]. While these studies implicate VRK3 in various disorders, they do not state the involvement of phosphoregulation, and demand future investigations.

## 8. Deciphering VRK3 Signaling Architecture: Pathway Convergence and Regulatory Functions

Through peptide library screening approach for kinase binding, LATS1 was identified as an upstream kinase that can phosphorylate VRK3 (S82) [44]. The Hippo signaling pathway, which controls organ size, apoptosis, and cell proliferation, is largely dependent on LATS1. The discovery of LATS1 suggests that VRK3 may be functionally controlled through the dynamics of the Hippo pathway, implying a potential crosstalk between Hippo signaling and VRK3-mediated regulation. Functioning as a key modulator of ERK signaling, VRK3 attenuates ERK activity by directly interacting with the mitogen-activated protein kinase phosphatase, VHR/DUSP3, which inactivates ERK within the nucleus [8].

VRK3 plays a pivotal role as a scaffold and regulatory protein [2,60]. It serves as a nuclear modulator of the ERK signaling cascade by directly interacting with the phosphatase VHR, enhancing its activity and thereby suppressing ERK phosphorylation and downstream signaling [2,8,61]. The balance between VRK3 and ERK activity is crucial for maintaining controlled cell growth and differentiation. Moreover, emerging evidence indicates that VRK3 contributes to chromatin organization and transcriptional regulation through interactions with histones and epigenetic factors such as JARID2 [6]. Its inhibition results in G1 cell-cycle arrest and metabolic alterations in glioma [7], while higher VRK3 levels has been associated with improved patient survival in head and neck squamous cell carcinoma [62]. Collectively, these findings establish VRK3 with potential implications in cancer biology and therapeutic targeting. Although VRK3 has been linked to multiple pathways, the co-differential phosphoregulatory network identified in this study provides a framework for understanding how VRK3 regulation is integrated across pathways and biological processes.

Experimental validation of the interactions and co-regulation patterns interpreted in this study remains to be performed to establish their functional relevance in specific disease contexts. Nevertheless, our integrative phosphoproteomics approach has provided novel insights into VRK3 phosphorylation and its associated regulatory network. We believe that these findings will serve as a valuable foundation for future wet-lab investigations aimed at validating the phosphorylation-dependent interactions identified here.

## 9. Conclusions

In this study, we present a comprehensive phosphoproteomic analysis of VRK3, a member of the VRK family. By leveraging large-scale mass spectrometry datasets, 32 phosphorylation sites on canonical isoform of VRK3, including S59, S82, and S83, were identified as the predominant sites. Integrative analysis of the kinase-substrate dataset derived from PSAP analysis revealed a set of 33 common upstream kinases, suggesting a conserved regulatory axis governing VRK3-associated phosphorylation events. Importantly, the identification of co-differentially phosphorylated VRK3 interactors highlights its broader involvement in synchronizing multiple signaling pathways, including Hippo, ERK, and GPCR-mediated networks. These findings position VRK3 as an active participant rather than a passive scaffold, with implications in cancer biology, neurodegeneration, and cell cycle regulation. Prospectively, investigations should focus on experimentally confirming the predicted upstream kinases and defining the functional significance of VRK3 phosphorylation sites via mutagenesis and biochemical assays. Exploring VRK3’s context-dependent functions in disease models, especially glioma and hepatocellular carcinoma, could reveal its utility as a diagnostic marker or therapeutic target. Emerging approaches such as single-cell phosphoproteomics and spatial proteomics may also provide greater resolution into VRK3’s signaling roles across different tissues. Collectively, current research on VRK3 phosphorylation sets the stage for advancing our understanding of pseudokinase biology and leveraging VRK3 as a key regulator within intricate cellular signaling networks.

## Data Availability

The datasets generated in this study are accessible through the Supplementary Materials.

## Supplementary Materials

The following supporting information can be downloaded at: https://www.mdpi.com/article/10.3390/proteomes14010014/s1, Supplementary Table S1: VRK3 Profoling data; Supplementary Table S2: VRK3 Differential Data; Supplementary Table S3: Proteins co-regulated with the major phosphorylation sites of VRK3; Supplementary Table S4: Upstream kinases that phosphorylate major phosphorylation sites of VRK3.
